# PRESS survey: PREvention of surgical site infection—a global pan-specialty survey of practice protocol

**DOI:** 10.3389/fsurg.2023.1251444

**Published:** 2023-09-25

**Authors:** J. Heinz, J. Walshaw, J. Y. Kwan, J. Long, D. Carradice, J. Totty, K. M. Kontouli, P. Lainas, L. Hitchman, G. Smith, B. Huo, H. Guadalajara, D. Garcia-Olmo, D. Sharma, C. S. Biyani, J. Tomlinson, M. Loubani, R. Galli, R. Lathan, I. Chetter, M. Yiasemidou

**Affiliations:** ^1^ Clinical Sciences Centre, Hull York Medical School, Hull, United Kingdom; ^2^ Academic Vascular Surgical Unit, Hull University Teaching Hospitals NHS Trust, Hull, United Kingdom; ^3^ Department of Health Sciences, University of York, York, United Kingdom; ^4^ Leeds Vascular Institute, Leeds Teachings Hospitals NHS Trust, Leeds, United Kingdom; ^5^ Department of Plastic and Reconstructive Surgery, Hull University Teaching Hospitals NHS Trust, Hull, United Kingdom; ^6^ Department of Mathematics, University of Ioannina, Ioannina, Greece; ^7^ Department of Digestive Surgery, Metropolitan Hospital, HEAL Academy, Athens, Greece; ^8^ Department of Minimally Invasive Digestive Surgery, Antoine-Béclère Hospital, Partis-Saclay University, Clamart, France; ^9^ Faculty of Medicine, Dalhousie University, Halifax, NS, Canada; ^10^ Department of Surgery, Hospital Universitario Fundación Jiménez Díaz, Universidad Autónoma de Madrid, Madrid, Spain; ^11^ Department of Surgery, Government NSCB Medical College, Jabalpur, India; ^12^ Department of Urology, St James University Hospital, Leeds Teaching Hospitals NHS Trust, Leeds, United Kingdom; ^13^ Trauma and Orthopedics, Sheffield Teaching Hospitals NHS Foundation Trust, Sheffield, United Kingdom; ^14^ Department of Cardiothoracic Surgery, Hull University Teaching Hospitals NHS Trust, Hull, United Kingdom; ^15^ Department of Surgery, Cantonal Hospital Baselland, Liestal, Switzerland; ^16^ Department of General Surgery, Bradford Teaching Hospitals NHS Trust, Bradford, United Kingdom

**Keywords:** survey, surgical wound infection, surgery, practice, guidelines

## Abstract

**Background:**

Surgical site infections (SSI) complicate up to 40% of surgical procedures, leading to increased patient morbidity and mortality. Previous research identified disparities in SSI prevention guidelines and clinical practices across different institutions. The study aims to identify variations in SSI prevention practices within and between specialties and financial systems and provide a representation of existing SSI preventative measures to help improve the standardization of SSI prevention practices.

**Methods:**

This collaborative cross-sectional survey will be aimed at pan-surgical specialties internationally. The study has been designed and will be reported in line with the CROSS and CHERRIES standards. An international study steering committee will design and internally validate the survey in multiple consensus-based rounds. This will be based on SSI prevention measures outlined in the CDC (2017), WHO (2018), NICE (2019), Wounds UK (2020) and the International Surgical Wound Complications Advisory Panel (ISWCAP) guidelines. The questionnaire will include demographics, SSI surveillance, preoperative, peri-operative and postoperative SSI prevention. Data will be collected on participants' surgical specialty, operative grade, of practice and financial healthcare system of practice. The online survey will be designed and disseminated using Qualtrics^XM^ Platform™ through national and international surgical colleges and societies, in addition to social media and snowballing. Data collection will be open for 3 months with reminders, and raking will be used to ascertain the sample. Responses will be analyzed, and the chi-square test used to evaluate the impact of SSI prevention variables on responses.

**Discussion:**

Current SSI prevention practice in UK Vascular surgery varies considerably, with little consensus on many measures. Given the inconsistency in guidelines on how to prevent SSIs, there is a need for standardization. This survey will investigate the disparity in SSI preventative measures between different surgical fields and countries.

## 1. Introduction

Over 300 million surgical procedures are performed annually worldwide (1). Up to 40% of surgical procedures are complicated by surgical site infections (SSI) (2–4). SSI rates vary by specialty, procedure, duration of the procedure and category of urgency. This variation may be due to differences in patient demographics across categories or underlying etiology of infection (5–7). Largely, SSI rates within RCTs report substantially higher incidences than nationally collected registries, owing to surveillance and diagnostic challenges within clinical practice. They are responsible for a substantial clinical burden, equating to one-third of all hospital-acquired infections (5). SSIs result in increased morbidity and mortality, with a 98% increased length of stay and a four-fold increased risk of readmission after discharge (3). Further, SSIs have a significant negative impact on the quality of life of patients, causing pain, immobility and psychological distress (8). SSIs require antimicrobial treatment, which can contribute to the development of antibiotic resistance (9). In addition, SSIs are associated with substantial healthcare costs due to protracted hospital stays, readmission, reoperation, pharmacological treatments, complex wound management systems and increased demands on staff resources (10). The true financial cost of SSI is likely to be underestimated due to wound surveillance challenges and limited access to outpatient services. However recent estimates suggest the cost to the NHS per infection is over £6103 (11).

In recent years, guidance on the prevention of SSI has been published by key organizations; National Institute for Health Care and Excellence (NICE), Centers for Disease Control and Prevention (CDC) and the World Health Organization (WHO) (12–14). However, a national survey of UK Vascular surgeons conducted by our group identified disparity in recommendations across these guidelines, which was noted to be due to the lack of underlying evidence (15). This study found variability in SSI prevention practices across different institutions, as well as a lack of relevant registries, clinical perception and literature data for SSI rates (15). This follow-on survey aims to assess the barriers in establishing uniform practice including the development of registries. As wound infections can occur after every type of surgery, wherever in the world it may take place, the steering committee of the current project decided to disseminate the follow-on survey to surgeons from all specialties internationally.

## 2. Methods

### 2.1. Objectives

Primary objective:

- To identify the barriers to establishing standardized SSI prevention practice.

Secondary objectives:

- To identify intra- and inter-specialty variations in surgical site infection prevention practice.
- To identify variations in practice amongst financial systems such as state and privately funded healthcare systems and economical classifications.

### 2.2. Study design

This is an international, pan-specialty, collaborative cross-sectional survey. A global panel of experts will form the study steering committee (SSC), providing a consensus-based approach to survey development, validation, and distribution. This study will be reported in line with the checklist for reporting of survey studies (CROSS) and the checklist for reporting results of internet E-surveys (CHERRIES) (16, 17). Ethical approval is provided by the Hull York Medical School Ethics Committee (REF22-23 10).

To collect data, the survey will be delivered electronically online using the Qualtrics^XM^ Platform™, Utah, USA. The format will be designed to include a combination of binary, Likert or multi-select. Free text comments will be used to collect further detail but will be kept to a minimum to help ensure good completion rates.

### 2.3. Questionnaire development

A pilot questionnaire will be developed based on SSI prevention measures outlined in the CDC (2017), WHO (2018), NICE (2019), Wounds UK (2020) and the International Surgical Wound Complications Advisory Panel (ISWCAP) guidelines. Additionally, feedback from the survey of surgical site infection prevention practice in UK vascular surgery will be used to inform survey questions/design (12–14, 18, 19). Since the survey will be targeting participants across multiple specialties, it will only contain general prevention measures widely applicable. A draft survey will be provided in Supplementary Material 1.

The questionnaire will be structured into five sections; demographics, SSI surveillance, preoperative, peri-operative and postoperative SSI prevention domains. Data will be collected on participant surgical specialty, operative grade, country of practice and financial healthcare system of practice. SSI data will include criteria used for diagnosis. There were 15 perioperative domains formulated the UK questionnaire, which will be scrutinized against the CDC, WHO, NICE and ISWCAP guidelines outlined above by the SSC, to form a pilot questionnaire. The process of questionnaire development will be carried out through discussion by members of the SSC and a record of any question refinement will be documented. The number of questions will not be rigidly defined but the SSC will consider the impact on survey duration which can influence completion rates.

The pilot questionnaire will be distributed to at least two consultants of each surgical specialty; vascular, general, orthopedic and trauma, urology, plastic and reconstructive, cardiothoracic, neurosurgery and otolaryngology for multiple rounds of validation. After each round feedback will be analyzed by the SSC and consensus-based changes will be adopted. Any alterations will be done so on unanimous decision and documented as major (questions removed or added), and minor (wording alterations). The survey will be validated further in subsequent rounds until the SSC agrees no further alterations are required.

### 2.4. Sample characteristics

The survey will be sent out electronically to surgeons of any specialty and at all levels of training worldwide. This includes consultants, and surgical trainees (specialties registrars/residents). To be included in the study, surgeons must be currently practicing and be registered with the national surgical body/college/society. The validated online survey will be distributed through Qualtrics^XM^ for single-stage cluster sampling in addition to dissemination through social media, where snowballing may occur. Each network of distribution will provide a cluster, i.e., VERN for the vascular surgeons and PIACO group for general surgery. The study population will provide a representation of existing SSI preventative measures used worldwide.

Given the global nature of the survey, no sample size will be calculated. Raking will be used to assign weight values to each survey respondent in such a manner, that the weighted distribution of the sample is in very close agreement with two or more marginal control variables. Socio-economic (developing vs. developed countries) variables and surgical specialties will be used to weigh the sample of responses. As such, results will be able to be extrapolated into wider populations, irrespective of the distribution of responses received.

### 2.5. Survey administration

The survey will be advertised in weekly rounds, 1 month prior to dissemination using affiliated society social media accounts and the Surgical Infection Research Network Twitter account (@SIRNglobal).

The survey will be disseminated worldwide, with an international SSC to establish a network of distribution. The committee has been involved in previous successful surveys and collaborative projects through the PIACO group, the Vascular Endovascular Research Network (VERN), James Lind Alliance (JLA) and ISWCAP surveys (20–23). The committee will also identify new routes of distribution within this project. The validated online survey will be distributed using an electronic link. Participants will receive an email via their affiliated membership organization, inviting them to take part and will include a direct link to the online survey. Following the invitation, surgeons will have 3 months to complete the survey, with reminders sent every 2 weeks. The survey will also be promoted via social media platforms, using the SIRN Twitter account in addition to affiliated organization accounts. The “prevent multiple submissions” option in Qualtrics will be enabled to prevent multiple participation of participants. A secondary IP address check will follow to ensure there are no remaining duplicate entries. No two entries from the same IP address will be allowed within 24 h. After the data collection window, the survey will lock out, preventing further responses. Participants will be offered an opportunity to win a £20 Amazon voucher as an incentive to participate in the survey. Survey items will not be randomized to improve participation, as the logical order of each survey section was unanimously agreed by the SSC to improve the response flow. Adaptive questioning will be used depending on the following questions from a response. The number of items and screens or pages on mobile and personal computers will be reported in the manuscript write-up.

## 3. Discussion

Current SSI prevention practice in UK Vascular surgery varies considerably, with little consensus on many measures (15). SSI prevention guidelines recommended by international bodies (12–14), include over 15 generic methods of preventing SSIs. Some of them are supported by evidence from randomized control trials (RCTs), such as avoiding razors for preoperative hair removal and the decolonization with intranasal antistaphylococcal agents for high-risk procedures. Both methods have been shown to reduce the SSI rate. The use of the WHO surgical checklist leads to a lower SSI rate after its implementation. The exact mechanism of this is suspected to be multifactorial (6). Multiple guidelines (12–14) recommend using antiseptic skin agents, though specific recommendations vary. Using alcohol-based chlorhexidine reduces the risk of SSI compared with aqueous iodine (24). Body surface warming systems to maintain normothermia perioperatively have strong evidence in preventing complications of hypothermia and lowering SSI rates (25). Postoperative negative pressure wound therapy decreases SSI rates in vascular surgeries but was not associated with a statistically significant decrease in SSI rates in other surgical disciplines (6). Antibiotic prophylaxis is recommended by all guidelines (12–14), RCTs focusing on vascular surgery demonstrated a significant reduction in SSI rate (26, 27). Although there are no RCTs on the effect of perioperative glycemic control and its impact on SSI rates, this is recommended in all major guidelines (6, 12–14). Retrospective studies do however confirm a higher risk of SSI rates in patients with postoperative hyperglycemia (6).

The incidence of SSI varies across surgical procedures, specialties, and conditions. Diagnostic and reporting challenges make true SSI rates difficult to capture. SSI rates are reported to vary from 0.1% to 40%. There are several patient- and procedure-related factors that influence the incidence of SSI. Patient comorbidities, advanced age, frailty and surgical complexity can increase the risk of developing an SSI. Additionally, prolonged duration and classification of the surgery are important procedure-related factors (28).

Given the inconsistency in guidelines on how to prevent SSIs, there is a need for standardization. This survey will investigate the disparity in SSI preventative measures between different surgical fields and countries. It will ascertain whether surgeons could feasibly participate in recruiting to platform randomized controlled trials assessing multiple interventions, including antibiotic prophylaxis.

### 3.1. Limitations

Reminders will be sent on a 2-weekly basis to increase the response rate and to prevent sampling bias. Additionally, the study will be advertised on its own Twitter account to gain attention. Response fatigue is a common issue, leaving some questions unanswered. To keep this to a minimum, the survey has been designed to only ask relevant questions and to be succinct, clear, and unambiguous.

Due to the nature of the study, there is room for responder and recall bias. To minimize this, questions wording and survey length have been considered carefully during all steps of the development of the survey. Questions will be categorized into SSI-related preoperative, perioperative, and postoperative themes to preserve the structure and enable ease of organization for respondents to follow.

Additionally, there may be discrepancies in the response rates between different countries, not providing an accurate representation of certain regions. There may also be different local/national guidelines and unequal access to certain resources depending on the geographical regions. As this survey will be delivered worldwide, questions are based on guidelines from the National Institute for Health and Care Excellence (NICE), Centers for Disease Control and Prevention (CDC) and World Health Organization (WHO). These guidelines are internationally recognized and available, therefore discrepancies between geographical regions and differences in local guidelines should be kept to a minimum.

There is no standardized consensus recommendation on SSI prevention regarding perioperative practice. Unsurprisingly this can lead to a discrepancy in clinical practice, as shown by a questionnaire study within the UK (15). If international and pan-surgical SSI prevention practices also vary, guidance recommended by international bodies is not being followed. This may be due to the lack of underlying evidence for SSI prevention practice, and thereby the necessity for high-quality RCTs to establish the best practice for patients and surgeons worldwide.
