# Supplementary material for: PRESS survey: PREvention of surgical site infection—a global pan-specialty survey of practice protocol
Source: Front Surg. 2023 Sep 25;10:1251444. doi: 10.3389/fsurg.2023.1251444 (PMC10560728; doi:10.3389/fsurg.2023.1251444)
Supplement: Supplementary file 1 [file Datasheet1.docx]

Supplementary Material

PRESS Survey: PREvention of Surgical Site Infection – A Global Pan-Specialty Survey of Practice Protocol

**Heinz J^1 2^ (†), Walshaw J^1 2 3^, Kwan JY^4^, Long J^1 2^, Carradice D^1 2^, Totty J^1 5^, Kontouli KM^6^, Lainas P^7 8^ , Hitchman L^1 2^, Smith G ^1 2^, Huo B^9^, Garcia-Olmo D^10^, Sharma D^11^, Biyani S^12^, Tomlinson J^13^, Loubani M^14^, Galli R^15^, Lathan R^1 2 3 *^ (†), Chetter I^1 2^, Yiasemidou M^1 16^**

*** Correspondence:** Lathan Ross: [ross.lathan2@nhs.net](mailto:ross.lathan2@nhs.net)

# Supplementary Data

Follow the link below or scan the QR code to view a draft version of the survey.

<https://york.qualtrics.com/jfe/form/SV_eE5tzjo7IOabTAG>


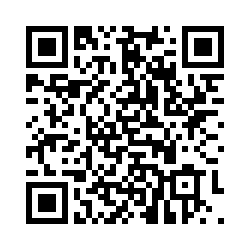


**
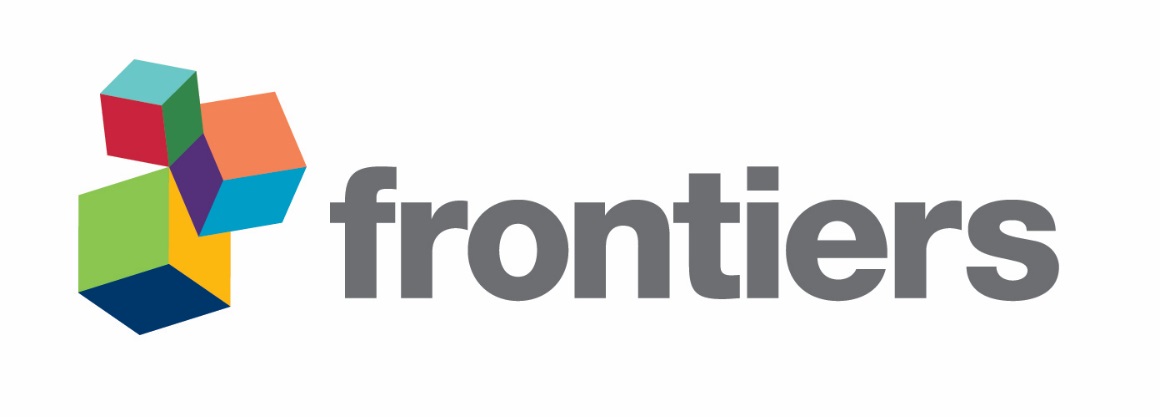
**
